# Freezing–Thawing Procedures Remodel the Proteome of Ram Sperm before and after In Vitro Capacitation

**DOI:** 10.3390/ijms20184596

**Published:** 2019-09-17

**Authors:** Patricia Peris-Frau, Alicia Martín-Maestro, María Iniesta-Cuerda, Irene Sánchez-Ajofrín, Lourdes Mateos-Hernández, J. Julián Garde, Margarita Villar, Ana Josefa Soler

**Affiliations:** 1 SaBio IREC (CSIC-UCLM-JCCM), ETSIAM, Campus Universitario s/n, 02071 Albacete, Spain; Patricia.Peris@uclm.es (P.P.-F.); Alicia.MartinMaestro@uclm.es (A.M.-M.); Maria.iniestacuerda@uclm.es (M.I.-C.); Irene.ssanchez@uclm.es (I.S.-A.); Julian.Garde@uclm.es (J.J.G.); MargaritaM.Villar@uclm.es (M.V.); 2 UMR BIPAR, INRA, Ecole Nationale Vétérinaire d’Alfort, ANSES, Université Paris-Est, 94700 Maisons-Alfort, France

**Keywords:** capacitation, cryopreservation, proteome, ram sperm

## Abstract

Mammalian sperm must undergo a set of structural and functional changes collectively termed as capacitation to ensure a successful oocyte fertilization. However, capacitation can be compromised by cryopreservation procedures, which alter the proteome and longevity of sperm. To date, how the protein changes induced by cryopreservation could affect the acquisition of sperm fertilizing potential remains unexplored. The present study investigated the protein profile of ram sperm during in vitro capacitation before and after cryopreservation to elucidate the impact of cryopreservation on sperm capacitation at a molecular level. Fresh and cryopreserved ram sperm were incubated under capacitating (CAP) and non-capacitating (NC) conditions for 240 min. The sperm proteome of these four treatments was analyzed and compared at different incubation times using reverse phase liquid chromatography coupled to mass spectrometry (RP-LC-MS/MS). The comparison between fresh and cryopreserved sperm suggested that cryopreservation facilitated an apoptosis-stress response and redox process, while the comparison between sperm incubated in CAP and NC conditions showed that capacitation increased those biological processes associated with signaling, metabolism, motility, and reproductive processes. In addition, 14 proteins related to mitochondrial activity, sperm motility, oocyte recognition, signaling, spermatogenesis, and the apoptosis-stress response underwent significant changes in abundance over time when fresh and cryopreserved sperm incubated in CAP and NC conditions were compared. Our results indicate that disturbances in a ram sperm proteome after cryopreservation may alter the quality of sperm and its specific machinery to sustain capacitation under in vitro conditions.

## 1. Introduction

After ejaculation, mammalian sperm become capable of fertilization into the female reproductive tract through a time-dependent process called capacitation [1]. This process involves physical and biochemical changes, including variations in protein composition, that drive hyperactivation and acrosome reaction, two crucial steps for zona pellucida penetration and subsequent oocyte fertilization. At the molecular level, although capacitation has been correlated with a cAMP-dependent increase in tyrosine phosphorylation, little is known about the molecular mechanisms implicated in this process [2,3]. In recent years, the use of proteomics techniques has provided a better understanding of the molecular changes that take place in sperm, allowing for the identification of phosphorylated proteins and other proteins with crucial roles in sperm capacitation in human [4,5], mouse [6,7], boar [8,9], buffalo [10], and other species [11].

For decades, it has been largely accepted that mature sperm are transcriptionally and translationally inactive due to the high level of chromatin compaction [12,13]. However, some authors proposed a new viewpoint, where translation of new proteins from mRNA transcripts can take place during sperm capacitation [14,15,16]. Therefore, the acquisition of sperm function and their fertilizing ability depend on post-translational modifications of existing proteins [17], interaction with proteins present in the male and female reproductive tract [18], or probably even on the synthesis of new proteins [19].

Although sperm cryopreservation has become a relevant tool for the long-term preservation of male fertility and genetic improvements within the livestock industry, it has been widely reported that the quality and longevity of frozen-thawed sperm is adversely affected. Moreover, immediately after thawing, a subpopulation of surviving sperm undergoes capacitation-like changes (cryo-capacitation) [20]. As a result, cryopreserved sperm require less time to achieve capacitation than fresh sperm [21,22]. Comparative proteomics studies between fresh and cryopreserved sperm have been performed in several species to understand the molecular mechanisms of cryoinjury. Different authors proposed that protein degradation, carbonylation, and premature protein phosphorylation could be the main causes of sperm function impairment after freezing-thawing procedures [23,24]. These comparative studies have also demonstrated that cryopreservation alters the expression levels and functional state of many proteins related to motility, viability, acrosome reaction, mitochondrial activity, premature capacitation, sperm-oocyte binding, and apoptosis [25,26,27,28,29].

Recent findings in the proteomics field have provided novel information about capacitation and cryopreservation; however, to our knowledge, there are no published works that investigate the differential protein profile of sperm in both of these events simultaneously. Therefore, in the present study, a comparative proteomic analysis was conducted between fresh and cryopreserved ram sperm incubated under capacitating or non-capacitating conditions to explore protein changes during in vitro capacitation and cryopreservation at the same time. A comprehensive knowledge of the proteome of capacitated sperm before and after freezing may help to identify those proteins altered by cryopreservation that are indispensable for maintaining sperm functionality and fertility. This is particularly important regarding sheep, where freezing-thawing procedures need to be enhanced to improve the quality, lifespan, and fertilizing potential of cryopreserved sperm.

## 2. Results

### 2.1. Influence of Cryopreservation and Capacitation on Sperm Functionality

Some functional parameters differed significantly between fresh and cryopreserved sperm when an overall comparison was performed, irrespective of incubation time and media (Table 1). Cryopreservation induced a significant (*p* < 0.05) reduction of sperm motility (total and progressive) and mitochondrial activity. However, ROS production and the proportion of apoptotic sperm was higher (*p* < 0.05) in cryopreserved than fresh samples.

Incubation in different media also produced changes in sperm functionality regardless of the type of sample (fresh or cryopreserved) (Table 2). When fresh and cryopreserved sperm were incubated under capacitating conditions (CAP), tyrosine phosphorylation, mitochondrial activity, and sperm motility (total and progressive) were significantly greater (*p* < 0.05). Conversely, ROS levels were higher after incubating fresh and cryopreserved sperm under non-capacitating conditions (NC).

### 2.2. Protein Profile of Fresh and Cryopreserved Ram Sperm Incubated under Capacitating and Non-capacitating Conditions

**Figure 1:**
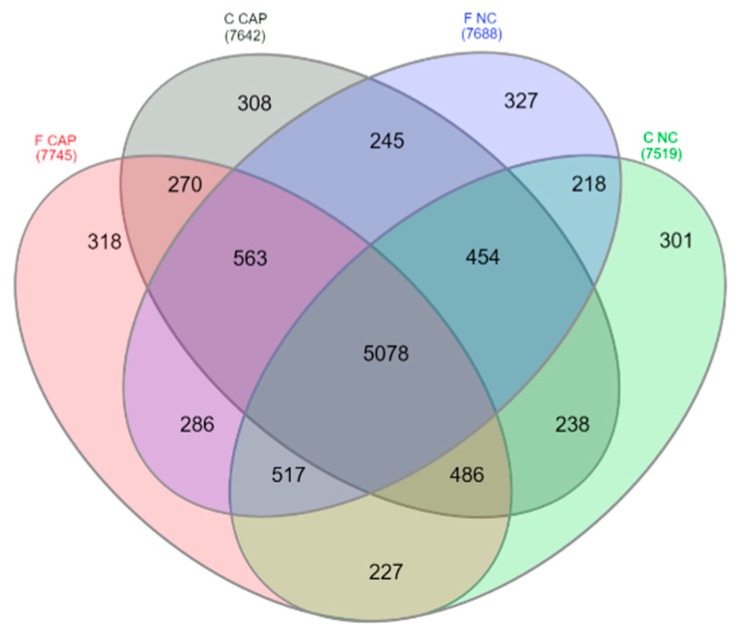
Venn diagram showing the distribution of ram sperm proteins between different treatments. F CAP and F NC represents the proteins detected in fresh sperm incubated under capacitating and non-capacitating conditions for all times, respectively; C CAP and C NC represent the proteins detected in cryopreserved sperm incubated under the same conditions for all times.

Proteomics analysis resulted in the identification of 10,899 proteins in fresh and cryopreserved sperm incubated in different media over time (Table S1). As expected, an important number of proteins (5078 proteins) were shared between fresh and cryopreserved sperm irrespective of incubation time and media (Figure 1). However, incubation in different media (CAP or NC) reduced the amount of common proteins between fresh and cryopreserved sperm in all times, sharing 270 proteins under CAP conditions and 218 proteins under NC conditions. 

### 2.3. Effect of Cryopreservation and Capacitation on Protein Changes

A statistical analysis between proteins identified in fresh and cryopreserved samples incubated under CAP and NC conditions at different time points was carried out to find out how cryopreservation and capacitating conditions affected ram sperm proteomes over time. After analysis, a total of 14 proteins showed quantitative differences (*p* < 0.05) (Table 3). Results were divided into two sections for a better understanding.

In the first section, the comparison of fresh and cryopreserved sperm samples at 0, 15, and 240 min in CAP and NC conditions (Table 3) revealed that six proteins were up-regulated and six were down-regulated in cryopreserved samples compared to fresh at different incubation times (0–15 min) in CAP or NC conditions. However, a prolonged incubation (240 min) in both media did not produce significant differences (*p* > 0.05) in protein levels between these two treatments. This data suggests that cryopreservation procedures remodeled the sperm proteome relatively fast. Most of the up-regulated proteins in cryopreserved sperm were located in the mitochondria and were involved in an apoptosis–stress response and several biological processes increased during sperm capacitation or cryo-capacitation, such as energy metabolism, motility, and oocyte recognition; meanwhile, most of the down-regulated proteins were located in the plasma membrane and were implicated in spermatogenesis and other relevant reproductive functions necessary for a successful fertilization event, such as oocyte recognition, signaling, and sperm motility, stressing the deleterious effects of cryopreservation (Table 3).

In the second section, the comparison of sperm samples immediately diluted in NC medium (0 min) with samples incubated in CAP medium at different time points (Table 3) showed that three proteins (mannose-6-phosphate/insulin-like growth factor II receptor (*M6P/IGF2R*); serine/threonine-protein phosphatase 2A alpha isoform (*PPP2R2A*); and dihydrolipoamide acetyltransferase component of pyruvate dehydrogenase complex (*DLAT*)) related to oocyte recognition, signaling, and metabolism were up-regulated (*p* < 0.05) in fresh sperm by the end of incubation period under CAP conditions, while in cryopreserved sperm, incubation under CAP conditions decreased (*p* < 0.05) the abundance of three proteins (hydroxyacyl-CoA dehydrogenase trifunctional multienzyme complex subunit alpha (*HADHA*); pituitary adenylate cyclase-activating polypeptide type 1 receptor hop 1 (*ADCYAP1R1*) and ankyrin repeat-SAM-basic leucine zipper domain-containing protein 1 (*ASZ1*)) involved in metabolism, motility, and spermatogenesis at different time points, but at the same time increased (*p* < 0.05) the abundance of another two proteins (*DLAT* and dolichyl-diphosphooligosaccharide-protein glycosyltransferase (*LOC101123268*)) related to oocyte recognition and metabolism at 15 min of incubation. These proteins were located in different regions of sperm to meet their specific functions (Table 3).

### 2.4. ELISA Analysis

**Figure 2:**
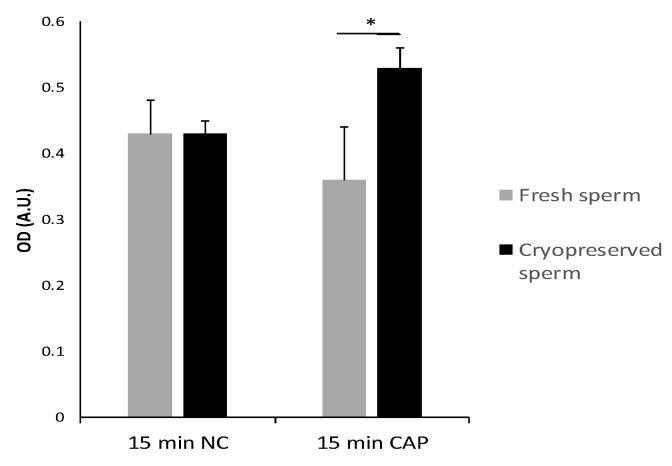
Optical densities (OD) of *CAPZA2* in fresh and cryopreserved sperm after 15 min of incubation in NC or CAP conditions assessed using ELISA. * *p* < 0.05.

Among the differentially abundant proteins, capping protein alpha 2, 3 prime (*CAPZA2*) was selected to validate the proteomics results due to the availability of a commercial antibody and its relevant role during in vitro capacitation. The increment of *CAPZA2* could be associated with the increase in actin polymerization reported during sperm capacitation since actin-binding proteins participate in the actin cytoskeleton organization [30]. The results of the ELISA analysis corroborated the proteomics results showing a higher (*p* < 0.05) protein concentration of *CAPZA2* in cryopreserved sperm at 15 min of incubation under CAP conditions in comparison with fresh sperm (Figure 2).

### 2.5. Functional Analysis of Identified Proteins in Fresh and Cryopreserved Sperm Incubated under Capacitating and Non-capacitating Conditions

**Figure 3:**
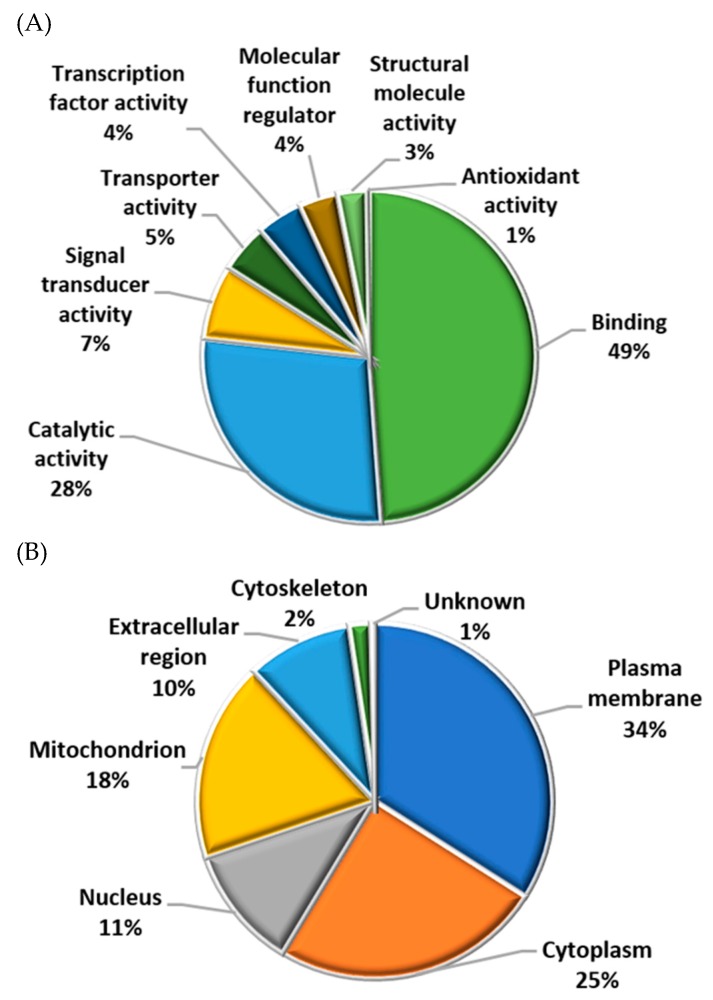

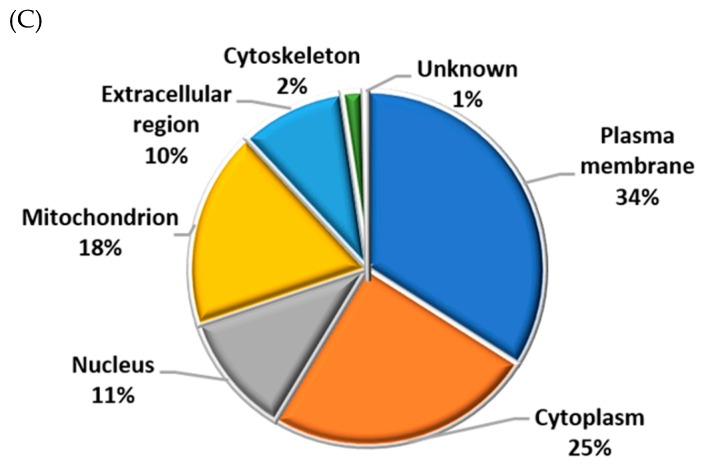
Classification of the identified proteins in fresh and cryopreserved ram sperm incubated in both media (CAP and NC) over time, according to their (**A**) molecular function, (**B**) subcellular location, and (**C**) biological process using Blast2GO and UniProtKB.

**Figure 4:**
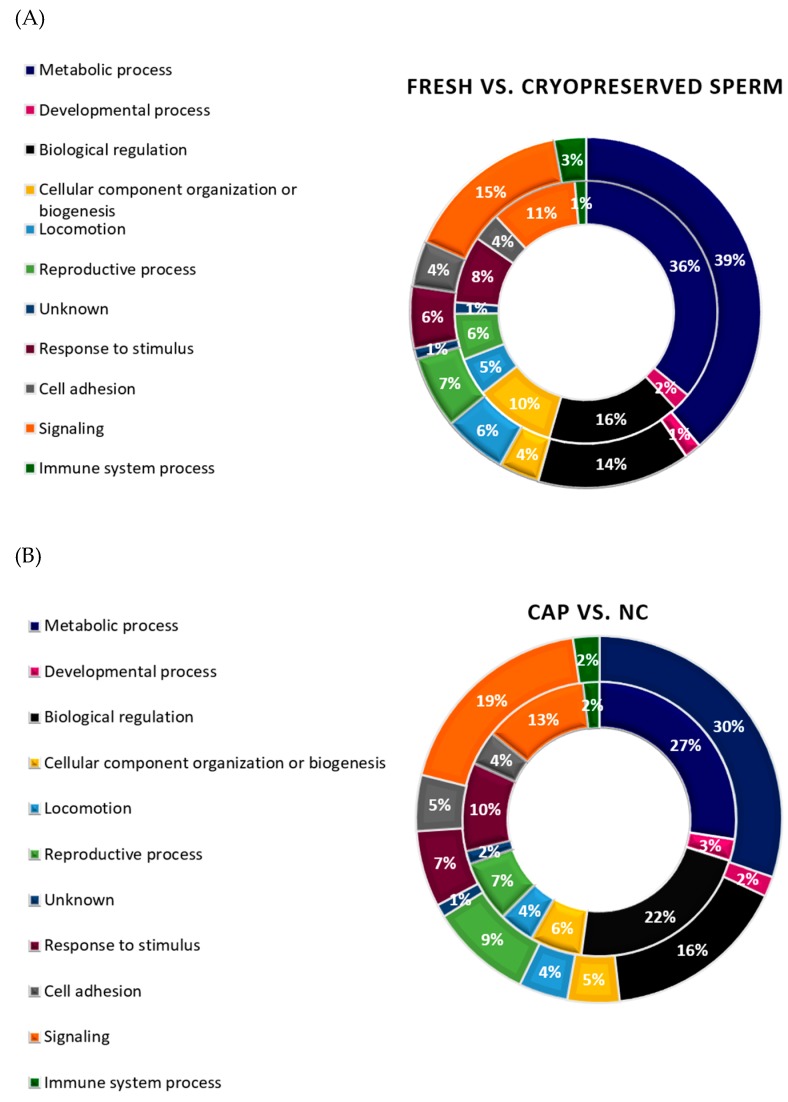
Biological process categorization of ram sperm proteins identified in different treatments. (**A**) Distribution of those proteins identified in fresh (outer circle) or in cryopreserved sperm (inner circle). (**B**) Distribution of those proteins present in sperm incubated in CAP (outer circle) or NC conditions (inner circle).

Most of the identified sperm proteins participated in molecular functions related to binding (49%) or catalytic activity (28%) (Figure 3A) and were located in the plasma membrane (34%), cytoplasm (25%), and mitochondria (18%) (Figure 3B). Regarding biological processes, most proteins were involved in cell metabolism, signaling, and regulation (Figure 3C and Figure 4). However, when biological processes were grouped for each treatment (fresh and cryopreserved sperm, sperm incubated in CAP and NC conditions), the protein distribution changed (Figure 4). Metabolism, signaling, locomotion, and reproductive processes were less represented in cryopreserved than fresh sperm (Figure 4A). But, biological regulation, cellular component organization, and response to stimulus were more represented after cryopreservation. A different protein distribution was also observed among sperm incubated in CAP or NC conditions (Figure 4B). Sperm capacitation in SOF-ESS raised the representation of diverse biological processes such as metabolism, signaling, reproductive, and cell adhesion processes, while biological regulation and other functions depicted in Figure 4B were reduced.

### 2.6. Different Representation of Those Biological Processes Directly or Indirectly Involved in Reproduction between Fresh and Cryopreserved Sperm Incubated under Capacitating and Non-Capacitating Conditions

**Figure 5:**
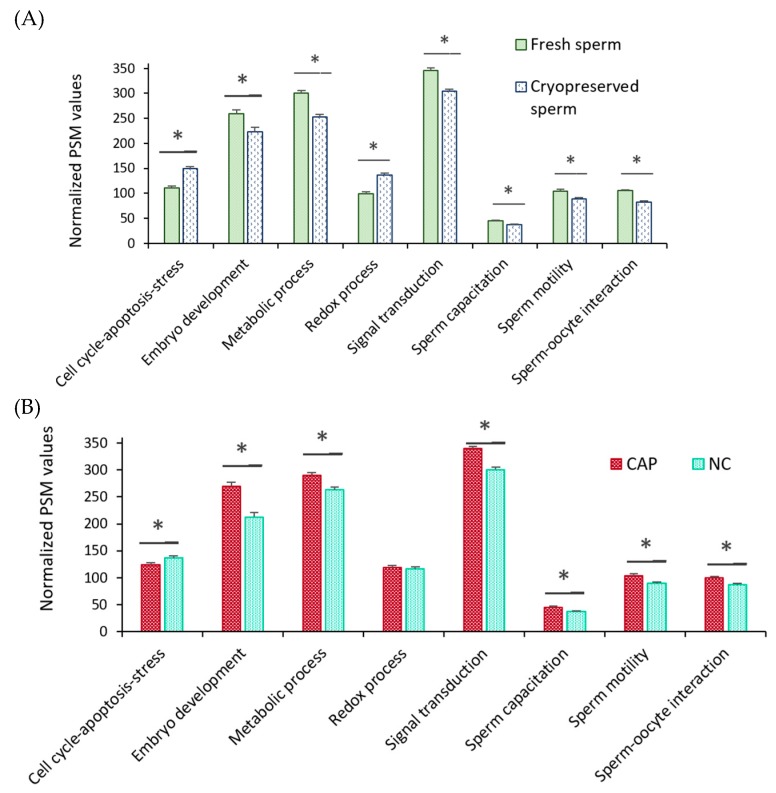
Different representations of an apoptosis-stress response and other biological processes directly or indirectly involved in reproduction between treatments. (**A**) Comparison of fresh versus cryopreserved ram sperm regardless of the incubation time and media. (**B**) Comparison of sperm incubated in CAP versus NC conditions, regardless of the incubation time and treatment (fresh or cryopreserved sperm). Peptide spectral matches (PSMs) of those proteins involved in each process were normalized against the total number of PSMs and compared using the Bonferroni test, * *p* < 0.05.

During sperm capacitation, a high supply of energy is required, increasing some metabolic processes. Moreover, signaling events, especially phosphorylation, and redox reactions also increase throughout capacitation. Therefore, these three biological processes, which are indirectly involved in reproductive functions and other reproductive processes were further evaluated and compared between different treatments to investigate how sperm cryopreservation and capacitation could affect them (Figure 5). Two comparisons were made; fresh versus cryopreserved sperm and sperm incubated in CAP versus NC conditions. When fresh and cryopreserved sperm were compared, regardless of the incubation time and media, fresh sperm had a higher (*p* < 0.05) representation of reproductive processes but a lower representation of those processes implicated in redox status and an apoptosis-stress response (Figure 5A). Meanwhile, when sperm incubated in CAP and NC conditions were compared without taking into account the type of sample (fresh or cryopreserved), embryo development, metabolism, sperm capacitation, sperm motility, cell signaling, and sperm-oocyte interaction were significantly (*p* < 0.05) more represented in those sperm incubated in CAP conditions than in NC (Figure 5B). Additional analyses (Table S2) revealed that those proteins associated with an apoptosis-stress response were more represented (*p* < 0.05) after a prolonged incubation in cryopreserved samples, while there was no change (*p* > 0.05) in representation in fresh samples over the incubation period. In addition, the latter samples required a longer incubation under CAP conditions to increase the representation of those proteins involved in several reproductive processes than the former (Table S2).

## 3. Discussion

In this study, we applied a proteomics approach to elucidate how cryodamage altered the response to capacitation in ram sperm. To the best of our knowledge, this is the first time that the proteome of fresh and cryopreserved sperm has been compared in sheep during in vitro capacitation over time.

Freezing-thawing procedures inflict drastic changes of temperature, osmolarity, and volume in sperm, as well as oxidative stress [31,32]. These stressful conditions promote a loss of cytoplasmatic or membrane proteins and conformational changes of some proteins due to denaturation, oxidation, or post-translational modifications [23,30,33], which cause biological process changes. As a result, we found that the proteins identified in cryopreserved ram sperm were more involved in those biological processes associated with redox status, biological regulation, cellular component rearrangement, and apoptotic-stress responses than the proteins identified in fresh sperm. These findings were supported by some changes observed in diverse functional sperm parameters when we compared fresh and cryopreserved samples. Moreover, as previous studies have reported [27,34,35,36], our results showed that cryopreservation induced increased ROS production, sperm motility reduction, impairment of mitochondrial activity, and increased the proportion of apoptotic sperm, probably due to mitochondrial oxidative damage. All the disturbances found in sperm functionality after cryopreservation and the minor participation of sperm proteins in reproductive processes suggest that the fertilizing ability of cryopreserved samples was reduced compared to fresh samples. In the latter samples, sperm were not subjected to the stressful conditions described before, which increased the representation of reproductive processes to meet the main function of these cells, which is a successful oocyte fertilization.

When fresh and cryopreserved ram sperm were incubated under CAP conditions, the biological processes associated with metabolism, signal transduction, and different reproductive functions, such as embryo development, sperm-oocyte interaction, motility, and sperm capacitation were more represented than in NC conditions. In line with these data, we found that incubation under CAP conditions increased sperm motility, mitochondrial activity, and tyrosine phosphorylation compared to NC conditions. During their journey through the female reproductive tract, sperm interact with different proteins, molecules, and hormones to achieve fertilization [18]. Under in vitro conditions, ram sperm are frequently incubated in a capacitating medium supplemented with estrous sheep serum (ESS) to acquire their fertilizing ability [37,38]. In our study, we employed SOF-ESS as a CAP medium, and since female reproductive fluids and serum share a similar composition [39], we hypothesized that some sperm proteins underwent conformational changes after interacting with several elements of ESS, which enhanced the representation of reproductive functions in capacitated sperm.

Apart from altering the biological processes distribution of many proteins and several functional sperm parameters, cryopreservation and in vitro capacitation of ram sperm changed the abundance of 14 proteins. The detrimental effects of cryopreservation were immediately observed on *TOPAZ1*, *M6P/IGF2R*, and *LOC101123268* after dilution in NC conditions. Changes in the sperm membrane architecture during cryopreservation due to cryodamage caused a protein reorganization, unmasking the expression of some proteins and promoting the loss of other cytoplasmatic or membrane proteins [28,40], which could explain the lower levels of *TOPAZ1*, a protein involved in spermatogenesis, and the different levels of those proteins involved in sperm-oocyte interactions (*M6P/IGF2R*, *LOC101123268*) in comparison with fresh sperm. According to Belmonte et al. [41], *M6P/IGF2R* might be involved in the recognition of specific glycoconjugates on the zona pellucida, mediating sperm-oocyte interactions and posterior binding. The higher levels of *M6P/IGF2R* at 0 min in cryopreserved sperm compared to fresh could be associated with capacitation-like changes that some sperm experience as a consequence of the increment in membrane permeability after cryopreservation [20,42]. In contrast, the abundance of *LOC101123268* at 0 min was lower in cryopreserved sperm than fresh sperm. This protein participates in N-glycosylation, a relevant post-translational modification associated with fertilization events, or more precisely, with gamete interaction and fusion [17,43]. In many cases, the loss or premature redistribution of membrane proteins implicated in oocyte recognition and fusion lead to fertilization failures [44], again explaining the reduced fertility of cryopreserved sperm in comparison with fresh sperm [31]. In addition, after 15 min under CAP conditions, the abundance of three proteins (*ADAMTS1*, *ADCYAP1R1*, and *RPN1*) decreased in cryopreserved samples compared to fresh samples. *ADAMTS1* has been associated with spermatogenesis, *ADCYAP1R1* with sperm motility, and *RPN1*, another glycosyltransferase like *LOC101123268*, with sperm-oocyte interaction [43]. The down-regulation of these proteins, together with the up-regulation of *PPP2R2B* in cryopreserved sperm after 15 min under CAP conditions in comparison with fresh sperm, suggest that proteins required for an optimal fertility and sperm function are being altered after freezing–thawing procedures, increasing the expression of proteins related to the apoptosis-stress response, such as *PPP2R2B*. In agreement with our findings, earlier studies demonstrated that cryopreservation increased the relative abundance of pro-apoptotic genes [36] and the expression of lncRNAs and mRNAs involved in apoptosis-related pathways [45].

Alternatively, the lower levels of *ADCYAP1R1* in cryopreserved samples at 15 min could indicate that this protein acted earlier during capacitation, rather than being a negative outcome of cryopreservation. *ADCYAP1R1* has been positively correlated with the increment of sperm motility in human sperm [46]. This protein might modulate sperm motility via its stimulatory effect on cAMP synthesis, which in turn, triggers protein kinase-A activation (PKA) and the subsequent phosphorylation reactions [47,48], which is the main regulatory pathway of sperm capacitation [49]. cAMP production rises to a maximum within 1 min, this time seems to be enough to activate PKA [50]; however, the activation of PKA has been reported to inhibit the activity of a soluble adenylyl cyclase [51]. It is possible that a similar situation occurred in our study with *ADCYAP1R1*, which explains its decrease in abundance at 15 min under CAP conditions.

Although some proteins seemed to be damaged by cryopreservation, other proteins like *DLAT*, *SUCLA2*, and *CAPZA2* showed higher levels in cryopreserved sperm after 15 min of incubation in CAP conditions compared to fresh sperm. *DLAT* and *SUCLA2* participate in the energy metabolism, where the former links the major energy sources in ram sperm [52], glycolysis and mitochondrial oxidative phosphorylation, via the conversion of pyruvate to acetyl-CoA in the tricarboxylic acid cycle, while the latter generates ATP in the tricarboxylic acid cycle [53]. To achieve the capacitated state, several metabolic processes, as well as their enzymes increase [54]. Therefore, the higher levels of both enzymes in cryopreserved sperm at early times may be a response to the increased demand of energy during capacitation. Moreover, a dynamic remodeling of the actin cytoskeleton occurs during sperm capacitation and acrosome reaction [55]. These phenomena involve actin polymerization and depolymerization, which is regulated by capping proteins like *CAPZA2*. The increment of these proteins could indicate that even though cryopreservation inflicted cellular damage, ram sperm were still able to attain capacitation at an accelerated rate. Surprisingly, after a long incubation period under CAP conditions (240 min), no changes in proteins content were detected between fresh and cryopreserved sperm. This data suggests that cryopreservation procedures altered the sperm proteome relatively fast.

We also observed that capacitation remodeled the proteome of fresh and cryopreserved sperm in a different manner over time. In fresh samples, a prolonged incubation time (240 min) under CAP conditions promoted an up-regulation of three proteins (*DLAT*, *M6P/IGF2R*, and *PPP2R2A*), while in cryopreserved samples, two proteins (*LOC101123268* and *DLAT*) were up-regulated after a short exposure to CAP conditions (15 min).

During capacitation, a high energy supply is required to initiate tyrosine phosphorylation and changes in the motility pattern known as hyperactivation [54], which could explain the increment of enzymes implicated in energy metabolism, such as *DLAT*. In addition, *DLAT* might also participate in ROS production [56]. These oxygen free radicals are important effectors of capacitation due to their stimulatory effect on the cAMP/PKA/tyrosine phosphorylation cascade [57], the main regulatory pathway of sperm capacitation, whose up-regulation, especially tyrosine phosphorylation, has been associated with the acquisition of hyperactivated motility and the preparation of sperm to undergo acrosomal exocytosis [49].

Despite protein phosphorylation being a well-characterized event during sperm capacitation [58,59], little is known about the role of protein phosphatases in this process. Therefore, the specific function of *PPP2R2A*, a serine/threonine phosphatase that regulates serine/threonine phosphorylation, is yet unclear [60], although a study in fowl sperm suggested that *PPP2R2A* could be involved in acrosome reaction [61].

Another relevant variation documented during capacitation is the reorganization of the sperm plasma membrane [62,63], which unmasks certain proteins, facilitating acrosome reaction and zona pellucida penetration to successfully fertilize the oocyte. Therefore, it is plausible that the presence of some membrane proteins related to the sperm-oocyte interaction increases once sperm are capacitated due to their redistribution in the plasma membrane, as has been reported with mannose-specific receptors [64]. This could be the case with *M6P/IGF2R* and *LOC101123268*, two membrane proteins implicated in oocyte recognition and fusion.

Considering the above-mentioned functions, all these proteins seem to play a pivotal role in sperm capacitation in both types of samples, which could indicate that fresh and cryopreserved sperm achieve their fertilizing potential at these incubation times. In line with our results, previous studies in ram sperm observed that cryopreserved sperm required shorter incubation times than fresh sperm to reach the capacitated state [22,65]. The mechanisms by which the expression of certain proteins increases remain unknown. Some studies suggest that mature sperm are able to synthesize new proteins that are indispensable for either the capacitation or fertilization process by mitochondrial-type ribosomes [14,15,66]. However, further investigations to confirm the existence of active mitochondrial-type ribosomes are necessary [67]. On the other hand, it is plausible that the interaction of sperm with the surrounding environment or extracellular vesicles increased the abundance of some proteins, as earlier studies have reported [28,40]. We speculate that, under CAP conditions, the interaction of sperm with ESS or its extracellular vesicles enhanced the expression of some proteins that are crucial for both capacitation and fertilization. In accordance with this hypothesis, Ferraz et al. [68] recently observed that incubation with oviductal extracellular vesicles enhanced the sperm quality and its fertilizing ability, possibly due to the interaction of the sperm proteome with these vesicles.

At the same time, incubation of cryopreserved sperm under CAP conditions also reduced the abundance of some proteins over time, such as *HADHA* and *ADCYAP1R1*. An excessive generation of ROS during cryopreservation contributes to lipid peroxidation increment [69], reducing the amount of unsaturated fatty acids present in the sperm membrane, which may explain the lower activity of enzymes involved in beta-oxidation, such as *HADHA*. This metabolic pathway, along with glycolysis and oxidative phosphorylation, produce the energy needed to fuel sperm motility [70,71]. In consequence, the down-regulation of *HADHA* through the time in cryopreserved sperm might be associated with the reduced motility observed in these samples compared to fresh samples. This hypothesis is supported by previous studies in which *HADHA* deficiency was correlated with a lower sperm motility in asthenozoospermic patients and with smaller litter sizes in pigs [72,73,74]. In this context, *HADHA* may be considered a potential fertility biomarker. The other protein, *ADCYAP1R1*, seems to regulate sperm motility via cAMP production and its reduced abundance at 15 min in the cryopreserved samples suggest again that *ADCYAP1R1* might act earlier during capacitation.

In conclusion, this study emphasizes those differential proteins altered during cryopreservation that are believed to play a key role during capacitation, being some of them identified for the first time in ram sperm in this study. Our results confirmed that freezing-thawing procedures produced substantial variations in the sperm proteome, having a deep impact on sperm quality and its specific machinery to sustain capacitation. Moreover, although further proteomics studies of ESS are needed, this fluid seems to remodel the proteome of ram sperm during capacitation, increasing the abundance of those proteins related to sperm-oocyte interaction, signal transduction, metabolism, and sperm motility.

## 4. Materials and Methods

### 4.1. Semen Collection and Initial Evaluation

Semen were collected via an artificial vagina from four Manchega rams (>3 years of age) that were housed at the experimental farm of University of Castilla-La Mancha. The procedures were carried out by the Reproduction Biology Group which is officially authorized for collecting and storing semen from sheep (ES07RS02OC) following the RD 841/2011. After collection, wave motion and individual sperm motility were evaluated subjectively using bright field microscopy and phase contrast microscope (Eclipse 50i Nikon; Tokyo, Japan), respectively. Only those ejaculates with wave motion values of 4 and sperm motility higher than 80% were selected for the study. Semen was mixed to eliminate variability between males.

### 4.2. Sperm Cryopreservation and Thawing

Each pool was divided into two fractions. One fraction was processed as fresh semen, while the other fraction was cryopreserved in a commercial freezing extender, Biladyl^®^ (Minitube; Tiefenbach, Germany) with 20% egg yolk and 7% glycerol. Briefly, semen was extended to 200 × 10^6^ sperm/mL in Biladyl^®^ and slowly cooled from 30 to 5 °C over 2 h. After 2 h of equilibration at 5 °C, semen were automatically packed into 0.25 mL straws and frozen in a programmable biofreezer (Planer Kyro 10 Series III; Planer PLC, London, United Kingdom) following a freezing curve (−20 °C/min from 5 °C to −100 °C and −10 °C /min from −100 °C to −140 °C). Cryopreserved semen were plunged into liquid nitrogen and stored in a liquid nitrogen container. Thawing was carried out by placing straws in a water bath at 37 °C for 30 s.

### 4.3. In Vitro Sperm Capacitation

Fresh and cryopreserved semen were centrifuged through single columns of Percoll^®^ 45% (Sigma-Aldrich; Madrid, Spain) (700× *g* for 10 min) to remove seminal plasma, bacterial contaminants, and extenders. Then, sperm pellets were diluted to 10 × 10^6^ sperm/mL and incubated for 240 min in capacitating and non-capacitating media at 38.5 °C under 5% CO_2_. To achieve sperm capacitation, fresh and cryopreserved sperm were incubated in synthetic oviductal fluid [75] (SOF) supplemented with 2% of estrous sheep serum (ESS; CAP). In small ruminants, the addition of ESS is indispensable to promoting acrosome reactions and optimize IVF outcomes [37,38,76]. Additionally, SOF supplemented with 0.1% polyvinyl alcohol (PVA) was employed as a non-capacitating medium (NC).

Capacitation status was confirmed by assessing the global increase in tyrosine phosphorylated proteins that characterizes this event [58,59]. Following the incubation of fresh and cryopreserved sperm in CAP or NC media, aliquots from different samples were centrifuged at 1000× *g* for 5 min. Then, sperm pellets were fixed in 100 µL of PBS with 2% (*v*/*v*) paraformaldehyde (Sigma-Aldrich; Madrid, Spain ) for 10 min at room temperature (RT), washed, and permeabilized with 0.1% (*v*/*v*) of BD FACS™ Permeabilizing Solution (BD Bioscience; Madrid, Spain) for 10 min at RT. After washing in PBS (5000× *g*; 5 min), the sperm pellets were resuspended in 100 µL of blocking buffer (10% (*w*/*v*) BSA in PBS) for 30 min at 38.5 °C. Samples were washed again and then incubated for 1 h at 38 °C with anti-phosphotyrosine monoclonal antibody Clone 4G10 (Millipore; Madrid, Spain) at a 1:300 dilution in the blocking buffer. Finally, sperm were washed and incubated with FITC-conjugated anti-mouse IgG antibody (Sigma-Aldrich; Madrid, Spain) at a 1:500 dilution in blocking buffer for 1 h in the dark at 38 °C. Negative controls were obtained via incubation with either the secondary antibody alone or with IgG1-FITC (isotype from murine myeloma, clone MOPC 21, Sigma-Aldrich; 1:500 dilution) instead of the primary antibody. Global protein tyrosine phosphorylation in different sperm samples was detected using a Flowsight^®^ flow cytometer (Amnis; Merck-Millipore, Madrid, Spain). At least 5000 sperm were recorded per sample. Results are shown in Figure S1.

### 4.4. Sperm Quality Assessment

Functional sperm parameters were evaluated after incubating fresh and cryopreserved sperm in CAP or NC media. Total and progressive motility were assessed with the Sperm Class Analyzer software (SCA^®^) (Microptic; Barcelona, Spain), as has been previously described by García-Álvarez et al. [37].

During apoptosis, mitochondrial activity and reactive oxygen species (ROS) production were measured using a flow cytometer (Flowsight^®^ controlled with the INSPIRE^®^ software). Samples were diluted to 5 × 10^6^ sperm/mL in different staining solutions prepared with SOF-PVA-HEPES and immediately handled, except those used for assessing mitochondrial activity (15 min of incubation in the dark at 37 °C). The staining solution for apoptosis was 12 µM of PI and 50 nM of YO-PRO-1; for mitochondrial activity, 25 nM of YO-PRO-1 and 100 nM of Mitotracker Deep Red; and for ROS production, 12 µM of PI and 5 µM of CM-H2DCFDA. YO-PRO-1+/PI− represented the proportion of apoptotic sperm, Mitotracker+/YO-PRO-1− represented viable sperm with active mitochondria, and ROS production was measured only in viable sperm (PI−). Five thousand sperm were recorded per sample and raw data were analyzed using the IDEAS^®^ software.

### 4.5. Protein Extraction

After incubation under CAP and NC conditions, the proteome of fresh and cryopreserved ram sperm was analyzed at 0, 15, and 240 min of incubation in NC conditions, and at 1, 15, and 240 min in CAP conditions. Sperm samples were centrifuged at 2000× *g* for 3 min and sperm pellets were washed twice with PBS at 7000× *g* for 2 min at 4 °C. The resulting pellets containing 50 × 10^6^ sperm/pellet were homogenized in 300 µL of lysis buffer (7 M urea, 2 M thiourea, 4% (*v*/*w*) CHAPS, 1% (*v*/*w*) protease inhibitor-cocktail, and 1% (*v*/*w*) phosphatase inhibitor-cocktail (Sigma-Aldrich; Madrid, Spain)). Samples were sonicated for 1 min in an ultrasonic cooled bath (USC100T, 230V/50Hz-60VA, VWR^®^; Madrid, Spain), followed by 10 s of vortexing. After 10 cycles of sonication-vortexing, samples were centrifuged at 15,000× *g* for 30 min at 4 °C to remove insoluble debris. Protein concentration of the supernatants was determined using a 2D-Quant Kit (GE Healthcare; Madrid, Spain), with BSA as the standard. All protein samples were stored in cryovials at −80 °C until further analysis.

### 4.6. Proteomics Data Acquisition and Analysis

Protein samples (100 μg/sample) were precipitated following a methanol/chloroform procedure [77]. After precipitation, protein extracts were resuspended, on-gel concentrated, and trypsin digested, and the resulting peptides were analyzed using RP-LC-MS/MS, as previously described by Villar et al. [78]. The MS/MS raw files were searched against the Uniprot-*Ovis aries* database (27,417 entries in March 2017) (available online: https://www.uniprot.org) using the SEQUEST algorithm (Proteome Discoverer 1.4, Thermo Fisher Scientific, San Jose, CA, USA). Search parameters included tryptic cleavage after Arg and Lys, up to two missed cleavage sites, with tolerances of 1 Da and 0.8 Da for precursor ions and MS/MS fragment ions, respectively, and Met oxidation and Cys carbamidomethylation were optionally allowed. Two biological replicates per sample were analyzed. A false discovery rate (FDR) < 0.05 was considered as the condition for successful peptide assignments, and the presence of at least two peptides per protein in at least one of the replicates analyzed were the necessary condition for protein identification (Table S1).

### 4.7. ELISA Validation

For validation of the proteomics results, CAPZA2 was selected among the differentially abundant proteins. This protein showed quantitative differences between fresh and cryopreserved sperm at 15 min in CAP conditions. Following the same procedure described for proteomics analysis, sperm proteins were extracted after 15 min of incubation in CAP or NC media. ELISA plates were coated with 100 μL (0.01 μg/μL solution of sperm proteins per well) of carbonate/bicarbonate buffer and incubated at 4 °C overnight. Then, 100 µL of blocking buffer (5% (*w*/*v*) skim milk powder in PBS) were added to each well and incubated for 1 h at RT, followed by four washes with PBS supplemented with 0.05% (*v*/*v*) Tween 20 (PBST) (Sigma-Aldrich; Madrid, Spain ). Samples were incubated for 1 h at 37 °C with rabbit polyclonal anti-CAPZA2 (ab175378; Abcam, Cambridge, UK) at a 1:2000 dilution in blocking buffer, followed by four washes with PBST. Finally, plates were incubated for 1 h at RT with goat anti-rabbit IgG H&L conjugated to peroxidase (HRP) (ab205718; Abcam) at a 1:1000 dilution in blocking buffer and subsequently washed with PBST four times. The color was developed via the addition of 100 μL of 3,3′,5,5′-tetramethylbenzidine (Promega Biotech; Madrid, Spain) and protected from the light for 20 min at RT. The optical densities were measured at 450 nm with an ELISA reader.

### 4.8. Gene Ontology Analysis

Functional annotations of identified proteins were carried out using Blast2GO software (version 3.0; available online: https://www.blast2go.com) and unannotated proteins were manually searched for in UniProtKB database (available online: http://www.uniprot.org). Sperm proteins were classified according to their gene ontology (GO) annotations for biological process, molecular function, and cellular component. GO annotations of proteins were grouped for each treatment (cryopreserved sperm, fresh sperm, sperm incubated in CAP medium, and sperm incubated in NC medium) to identify changes due to cryopreservation or capacitating conditions. Finally, special attention was paid to those proteins involved in an apoptosis-stress response and other biological processes that are directly or indirectly implicated in reproductive functions.

### 4.9. Statistical Analyses

The average number of peptide spectrum matches (PSMs) for each protein was calculated from the two replicates and then normalized against the total number of PSMs in each sample. A paired comparison χ^2^ test (*p* < 0.05) between different samples (0 F NC, 0 C NC, 1 F CAP, 1 C CAP, 15 F CAP, 15 C CAP, 15 F NC, 15 C NC, 240 F CAP F, 240 C CAP, 240 F NC, 240 C NC) was performed to quantify significant changes in protein abundance. IDEG6 software (available online: http://telethon.bio.unipd.it/bioinfo/IDEG6/) was used for this analysis. The protein concentration of CAPZA2 was compared between fresh and cryopreserved sperm at 15 min using Student’s *t*-test with unequal variance (*p* < 0.05) using SPSS v. 23.0 (IMB Corp., Chicago, IL, USA). Differences in sperm quality, global tyrosine phosphorylation, and reproductive-processes-distribution between samples were evaluated in the two biological replicates using a general linear model (GLM) with SPSS. Post hoc comparisons were carried out using the Bonferroni test after obtaining *p* < 0.05.

## Figures and Tables

**Table 1:** Functional parameters of ram sperm before and after cryopreservation. Data represent the average values of fresh or cryopreserved sperm incubated in both media and are expressed as means ± SEM.

Sperm Parameters	Fresh Sperm	Cryopreserved Sperm
Total motility (%)	44.16 ± 4.17	22.48 ± 3.89 *
Progressive motility (%)	29.48 ± 3.40	17.16 ± 3.25 *
Apoptosis (%)	12.05 ± 2.21	20.16 ± 1.35 *
Mitochondrial activity (%)	32.44 ± 2.45	18.64 ± 2.45 *
ROS levels (mean fluorescence intensity)	58.83 ± 4.32	86.82 ± 4.01 *
Tyrosine phosphorylation (%)	46.68 ± 5.07	57.11 ± 5.07

* Indicate significant differences (*p* < 0.05) among treatments.

**Table 2:** Functional parameters of ram sperm during incubation under capacitating (CAP) and non-capacitating conditions (NC). Data represent the average values of fresh and cryopreserved sperm incubated in CAP or NC conditions and are expressed as means ± SEM.

Sperm Parameters	CAP	NC
Total motility (%)	41.69 ± 4.50	24.92 ± 4.50 *
Progressive motility (%)	28.81 ± 3.48	17.85 ± 3.92 *
Apoptosis (%)	14.84 ± 1.54	16.37 ± 1.54
Mitochondrial activity (%)	31.68 ± 2.54	19.40 ± 2.67 *
ROS levels (mean fluorescence intensity)	56.29 ± 2.54	89.36 ± 3.10 *
Tyrosine phosphorylation (%)	64.73 ± 4.49	39.05 ± 5.01 *

* Indicate significant differences (*p* < 0.05) among treatments.

**Table 3:** Differentially abundant proteins (*p* < 0.05) in fresh (F) and cryopreserved (C) ram sperm incubated in CAP or NC conditions at different incubation times.

Accession Number	Protein Name	Gene ID	Protein Representation		Reproductive Process	Subcellular Location
			In Cryopreserved Sperm ^a^ (Fresh vs. Cryopreserved Sperm)	In CAP Conditions over Time ^b^ (0–240 min)		
E5FYH0	Testis- and ovary-specific PAZ domain containing protein 1	*TOPAZ1*	↓ at 0 min in NC		Spermatogenesis	Cytoplasm
Q8SQ25	Mannose-6-phosphate/insulin-like growth factor II receptor	*M6P/IGF2R*	↑ at 0 min in NC	↑ after 240 min in F	Sperm-oocyte interaction	Plasma membrane
B6UV59	Hydroxyacyl-CoA dehydrogenase trifunctional multienzyme complex subunit alpha	*HADHA*	↑ at 0 min in NC	↓ after 1 min in C	Metabolic process	Mitochondria
W5PEA2	Succinate-CoA ligase (ADP-forming) subunit beta, mitochondrial	*SUCLA2*	↑ at 15 min in CAP		Metabolic process	Mitochondria
W5P1S6	Dolichyl-diphosphooligosaccharide-protein glycosyltransferase subunit 1	*RPN1*	↓ at 15 min in CAP		Sperm-oocyte interaction	Plasma membrane
W5PJ95	Serine/threonine-protein phosphatase 2A 55 kDa regulatory subunit A, alpha isoform	*PPP2R2A*		↑ after 240 min in F	Signal transduction	Cytoplasm
W5PEC5	Dolichyl-diphosphooligosaccharide-protein glycosyltransferase	*LOC101123268*	↓ at 0 min in NC	↑ after 15 min in C	Sperm-oocyte interaction	Plasma membrane
W5QCD4	Ankyrin repeat-SAM-basic leucine zipper domain-containing protein 1	*ASZ1*		↓ after 240 min in C	Spermatogenesis	Cytoplasm
D5K281	ADAM metallopeptidase with thrombospondin type 1 motif 1	*ADAMTS1*	↓ at 15 min in CAP		Spermatogenesis	Plasma membrane
W5QBN6	Dihydrolipoamide cetyltransferase component of pyruvate dehydrogenase complex	*DLAT*	↑ at 15 min in CAP	↑ after 15 min in C; ↑ after 240 min in F	Metabolic process	Mitochondria
Q8WMQ9	Pituitary adenylate cyclase-activating polypeptide type 1 receptor hop 1 splice variant	*ADCYAP1R1*	↓ at 15 min in CAP	↓ after 15 min in C	Sperm motility	Plasma membrane
W5NZH7	Serine/threonine-protein phosphatase 2A 55 kDa regulatory subunit B	*PPP2R2B*	↑ at 15 min in CAP		Cell cycle-apoptosis-stress	Mitochondria
Q09YI7	Capping protein (actin filament) muscle Z-line, alpha 2, 3 prime	*CAPZA2*	↑ at 15 min in CAP		Sperm motility	Cytoskeleton
A0A0C5GE36	V-kit Hardy–Zuckerman 4 feline sarcoma viral oncoprotein	*KIT*	↓ at 15 min in NC		Signal transduction	Plasma membrane

^a^ Protein representation in cryopreserved sperm (↑ increased, ↓ decreased or-no differences) after being compared with fresh sperm at different incubation times (0, 15, and 240 min) in CAP or NC conditions. ^b^ Protein representation of fresh or cryopreserved sperm (↑ increased, ↓ decreased or-no differences), after comparing time 0 min with different incubation times (1 min, 15 min, and 240 min) under CAP conditions.

## Supplementary Materials

Supplementary materials can be found at https://www.mdpi.com/1422-0067/20/18/4596/s1.
