# Supplementary material for: Freezing–Thawing Procedures Remodel the Proteome of Ram Sperm before and after In Vitro Capacitation
Source: Int J Mol Sci. 2019 Sep 17;20(18):4596. doi: 10.3390/ijms20184596 (PMC6769739; doi:10.3390/ijms20184596)
Supplement: Supplementary file 1 [file ijms-20-04596-s001.zip › ijms-585304 Supplementary/Supplementary Figure 1.pdf]

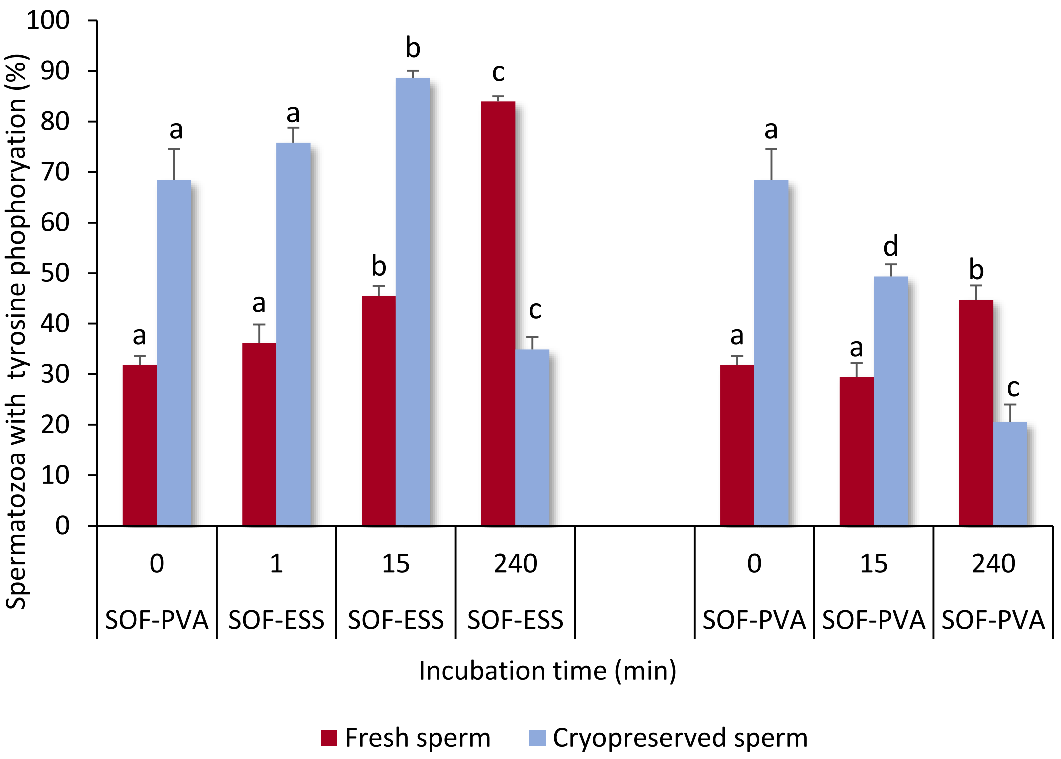

Figure S1. Dynamics of global protein tyrosine phosphorylation in ram sperm. Fresh and cryopreserved ram sperm were evaluated separately at multiple time points after incubation in SOF supplemented with estrous sheep serum (SOF-ESS) or in SOF supplemented with polyvinyl alcohol (SOF-PVA). The percentage of sperm showing protein tyrosine phosphorylation at each time was represented in the figure. Letters (a–d) illustrate significant differences ($p < 0.05$) during incubation period in both media in fresh or cryopreserved sperm.
