# Supplementary material for: Freezing–Thawing Procedures Remodel the Proteome of Ram Sperm before and after In Vitro Capacitation
Source: Int J Mol Sci. 2019 Sep 17;20(18):4596. doi: 10.3390/ijms20184596 (PMC6769739; doi:10.3390/ijms20184596)
Supplement: Supplementary file 1 [file ijms-20-04596-s001.zip › ijms-585304 Supplementary/Supplementary Table 2.pdf]

Table S2. Effect of incubation media and time on the distribution of those proteins related to reproductive processes and apoptosis-stress response in (**A**) fresh and (**B**) cryopreserved ram sperm. Peptide spectrum matches (PSMs) of those proteins involved in each process were normalized against the total number of PSMs. Data are expressed as means ± SD. Letters (a–d) represent significant differences (p < 0.05) in each process in cryopreserved or fresh sperm over time.

A)

	Fresh Sperm (Normalized PSMs Values)					
Reproductive Process	0 min	1 min	15 min		240 min	
	SOF-PVA	SOF-ESS	SOF-PVA	SOF-ESS	SOF-PVA	SOF-ESS
Cell cycle-apoptosis-stress	105 ± 8	106 ± 4	111 ± 1	1010 ± 3	111 ± 5	100 ± 10
Embryo development	201 ± 1[a]	253 ± 6[bc]	236 ± 2[c]	276 ± 3[b]	238 ± 5[c]	349 ± 4[d]
Metabolic process	257 ± 3[a]	313 ± 2[bc]	302 ± 7[bc]	317 ± 1[bc]	292 ± 10[c]	325 ± 1[b]
Signal transduction	231 ± 5[a]	347 ± 4[b]	332 ± 1[b]	382 ± 1[c]	352 ± 3[b]	432 ± 2[d]
Sperm capacitation	34 ± 2[a]	42 ± 1[ac]	41 ± 2[ac]	50 ± 1[bc]	43 ± 1[ac]	57 ± 1[b]
Sperm-oocyte interaction	93 ± 2[a]	104 ± 3[ab]	102 ± 2[ab]	108 ± 2[b]	106 ± 1[ab]	115 ± 1[b]

B)

	Cryopreserved Sperm (Normalized PSMs Values)					
Reproductive Process	0 min	1 min	15 min		240 min	
	SOF-PVA	SOF-ESS	SOF-PVA	SOF-ESS	SOF-PVA	SOF-ESS
Cell cycle-apoptosis-stress	146 ± 4[ac]	131 ± 2[a]	148 ± 6[ac]	128 ± 2[a]	176 ± 2[b]	165 ± 2[bc]
Embryo development	186 ± 1[a]	247 ± 2[b]	217 ± 4[c]	270 ± 4[b]	198 ± 6[ac]	221 ± 2[bc]
Metabolic process	233 ± 1[a]	263 ± 1[bc]	254 ± 2[ab]	280 ± 6[c]	240 ± 1[ab]	247 ± 6[ab]
Signal transduction	320 ± 4[ab]	289 ± 14[ac]	310 ± 1[ac]	374 ± 2[b]	260 ± 8[c]	273 ± 3[ac]
Sperm capacitation	32 ± 1[a]	41 ± 1[ab]	35 ± 1[a]	47 ± 2[b]	32 ± 1[a]	34 ± 3[a]
Sperm-oocyte interaction	73 ± 2[a]	91 ± 3[bc]	75 ± 2[a]	101 ± 5[b]	73 ± 6[a]	81 ± 2[ac]
